# Supplementary material for: Obstructions in Vascular Networks: Relation Between Network Morphology and Blood Supply
Source: PLoS One. 2015 Jun 18;10(6):e0128111. doi: 10.1371/journal.pone.0128111 (PMC4472785; doi:10.1371/journal.pone.0128111)
Supplement: S1 Supporting Information — (PDF) [file pone.0128111.s001.pdf]

Supplementary Material

Response function of a vessel network

We consider that the flow in each vessel of the network follows a dynamic Darcy's law in frequency domain [21], namely:

$$Q_i = -\frac{1}{\eta}\frac{A_i K_i}{l_i}\Delta p_i \ ,$$

where Q_i, A_i, K_i, l_i and Δp_i are respectively the flow, the cross sectional area, the dynamic permeability, the length and the pressure drop of the vessels at the i-th level.

Similarly, we write the total flow in a network as a dynamic Darcy's law:

$$Q = -\frac{1}{\eta}\frac{A_{eff} K_{eff}}{L}\Delta p \ .$$

We can define the response function of the network $\chi = A_{eff}K_{eff}$ as the product of an effective area, A_{eff}, and an effective permeability, K_{eff}. $\frac{L}{\chi}$ is the total resistance of the network.

In order to obtain the response function, χ, for the total network, we use an electrical analogy, where $R_i = \frac{l_i}{A_i K_i}$ is the resistance of each vessel. In this way, we assume mass conservation at every bifurcation, and a total pressure drop which is the sum of individual pressure drops. Therefore, Eq. (1) of the main text is obtained by interpreting the tree-like network as an association of resistances.

For an obstructed network, we consider that obstructions occur in half of the branches of the same tree level as illustrated in Fig. 1A of the main document. We consider a reduction of the cross sectional area in obstructed vessels at level n, which implies a different resistance, R_n^{obs}, for half of the vessels at that level. Using our electrical analogy (see Fig. 1B of the paper), the total resistance for a N-level network obstructed at level n is obtained through resistance association, yielding Eq. (3) of the main text.

In order to obtain the expressions for $\ln(\chi_{un} - \chi)$, Eqs. (4), (7)-(10), (12) and (13) of the main text, we take the following approximation:

$$\frac{L}{\chi} - \frac{L}{\chi_{un}} = L\,\frac{\chi_{un} - \chi}{\chi\,\chi_{un}} \approx L\,\frac{\chi_{un} - \chi}{\chi_{un}^2},$$

the difference of the responses for the unobstructed and obstructed networks reduces to:

$$\chi_{un} - \chi \approx \frac{\chi_{un}^2}{L}\left[\frac{L}{\chi} - \frac{L}{\chi_{un}}\right].$$

In the Following sections we obtain $\frac{L}{\chi} - \frac{L}{\chi_{un}}$ for the different networks studied in this work.

Network with a geometric progression of resistances

We now consider a progression of resistances in the network such that $a = R_i/R_{i-1}$ is constant. In this way, the resistance of a vessel in level i is given by

$$R_i = R_1 a^{i-1} \ ,$$

where R_1 is the resistance of the first level.

We can write the resistance of the obstructed vessel at level n as the product of a factor, $F > 1$, by the resistance of the unobstructed vessel at the same level, $R_n^{obs} = F\,R_n$. To linear order in ω

$$F = \frac{1}{(1-f)^2} + i\,\frac{f}{(1-f)^2}\,\frac{r_n^2\,\rho}{6\,\eta}\,\omega \ , \tag{1}$$

where f is the fraction of the total cross sectional area that has been obstructed. The two terms on the right hand side of this equation are obtained by expanding the permeabilities, $K_n^{obs} = K_n(r = r_n^{obs})$ and K_n, of the obstructed and unobstructed vessels for small values of the frequency. The analytical expression for K_n is given in terms of Bessel functions as explained in the main text after Eq. (1). Substituting the density ρ, frequency ω, and viscosity η of the dog blood on the second term of Eq. (1), we conclude that it is only relevant for large obstructions in vessels with diameter larger than 0.6 cm. Since this is rarely the case in the networks analyzed in the dog circulatory system (as well as in human vasculatures), we use the $\omega \to 0$ approximation and consider that the factor F takes the value $1/(1-f)^2$.

Substituting R_i and R_n^{obs} into the expression for the difference of the total resistances for the obstructed and unobstructed network (difference between the Eqs (3) and (1) of the manuscript), we obtain:

$$\frac{L}{\chi} - \frac{L}{\chi_{un}} = \frac{2R_1(F-1)\left[\left(\frac{a}{2}\right)^N - \frac{2}{a}\left(\frac{a}{2}\right)^n\right]}{2a\left(\frac{2}{a}\right)^n\left(\frac{a}{2}\right)^N + [a(F-1) - 2(F+1)]} \ . \tag{2}$$

In this expression, if $|a| < 2$, the second term inside the brackets in the numerator and the second term in the denominator dominate and we obtain Eq. (7) of the main text. In the particular case in which $a = 1$, Eq. (7) reduces to Eq. (4).

On the other hand, if $|a| > 2$, the first term inside the brackets in the numerator and the first term in the denominator dominate and we obtain Eq. (8) of the main text.

While in general a is a complex number, in the range of frequencies and vessel radii studied in this work, the imaginary part of a is always much smaller than its real part. This is the range of validity of Eqs. (7) and (8).

Network with a jump in vessel resistances

Finally, we consider a network for which the vessels have a resistance R_1 when $i \leq k$, and a resistance $R_2 = aR_1$ for $i > k$, where the level k is approximately at the middle of the network.

For obstructions at level $n \leq k$ we use R_1 and R_2 in Eqs. (1) and (3) of the main text and obtain:

$$\frac{L}{\chi} - \frac{L}{\chi_{un}} = \frac{2(F-1)R_1\left[2 + (a-1)2^{n-k} - 2^{n-N}a\right]}{(F+3) + 2^{n-k+1}(a-1) - 2^{n-N+1}a} \left(\frac{1}{2}\right)^n . \tag{3}$$

In this expression, the third term inside the brackets in the numerator, and the third term in the denominator are always much smaller than the corresponding second terms, so Eq. (3) reduces to:

$$\frac{L}{\chi} - \frac{L}{\chi_{un}} \approx \frac{2(F-1)R_1\left[2 + (a-1)2^{n-k}\right]}{(F+3) + 2^{n-k+1}(a-1)} \left(\frac{1}{2}\right)^n . \tag{4}$$

As it is described in the main text, for a large jump in resistance and/or if the obstructions are close from level k, $(a-1)\left(\frac{1}{2}\right)^{k-n} >> \frac{4-6f+3f^2}{(1-f)^2}$. In this case, the second term inside the brackets in the numerator and the second term in the denominator in Eq. (4) dominate. After making the corresponding approximation we obtain Eq. (12) of the main text.

In the situation that the jump in resistances is small, namely $(a-1)\left(\frac{1}{2}\right)^{k-n} << 1$, the first term of the brackets in the numerator and the first term in the denominator in Eq. (4) dominate. In this situation, we obtain Eq. (9) of the main text.

For obstructions at level $n > k$ we obtain a simpler expression:

$$\frac{L}{\chi} - \frac{L}{\chi_{un}} = \frac{4R_2(F-1)\left[1 - 2^{n-N-1}\right]}{(F+3) - 2^{n-N+1}} \left(\frac{1}{2}\right)^n . \tag{5}$$

In this expression, if the obstructions are not located in the last two levels, the second term of the brackets in the numerator and the second term in the denominator are much smaller than the first terms. In this case, this equation becomes:

$$\frac{L}{\chi} - \frac{L}{\chi_{un}} \approx \frac{4R_2(F-1)}{F+3} \left(\frac{1}{2}\right)^n \tag{6}$$

and we obtain Eqs. (10) and (13) of the main text.

It is relevant to note that for very large obstructions, i.e. for $F \to \infty$, Eqs. (4) and (6) can be rather simplified when written in the neighborhood of a large jump in resistance ($a \gg 1$). In this case,

$$\frac{L}{\chi} - \frac{L}{\chi_{un}} \approx \begin{cases} 4R_1 a 2^{-k-1} & n < k+1 \\ 4R_2 2^{-n} & n \geq k+1 \end{cases} . \tag{7}$$

This limit can be observed in figure 8B of the main text for 99.99 % of obstruction. Notice that the decrease in response described by Eq. (7) in this limit is continuous at $n = k + 1$, i.e., the expression yielded by the two branches of Eq. (7) have the same value at $n = k + 1$. Therefore close to vessel suppression, when the fraction of cross section obstruction approaches 100 %, the jump in $\Delta\chi$ becomes small and tends to zero, as described in the main text. This limit might not be physiologically relevant and it is mentioned for completeness of the mathematical analysis.
